# Climate Change and Effects on Molds and Mycotoxins

**DOI:** 10.3390/toxins14070445

**Published:** 2022-06-30

**Authors:** Veronica Zingales, Mercedes Taroncher, Piera Anna Martino, María-José Ruiz, Francesca Caloni

**Affiliations:** 1 Laboratory of Food Chemistry and Toxicology, Faculty of Pharmacy, University of Valencia, Av. Vicent Andrés Estelles, s/n, Burjassot, 46100 Valencia, Spain; vezin@uv.es (V.Z.); mercedes.taroncher@uv.es (M.T.); m.jose.ruiz@uv.es (M.-J.R.); 2 Laboratory of Toxicology, Faculty of Pharmacy, University of Valencia, Av. Vicent Andrés Estelles, s/n, Burjassot, 46100 Valencia, Spain; 3 Department of Biomedical, Surgical and Dental Sciences-One Health Unit, Università degli Studi di Milano, Via Pascal 36, 20133 Milan, Italy; piera.martino@unimi.it; 4 Department of Environmental Science and Policy (ESP), Università degli Studi di Milano, Via Celoria 10, 20133 Milan, Italy

**Keywords:** climate change, distribution, effects, molds, mycotoxins

## Abstract

Earth’s climate is undergoing adverse global changes as an unequivocal result of anthropogenic activity. The occurring environmental changes are slowly shaping the balance between plant growth and related fungal diseases. Climate (temperature, available water, and light quality/quantity; as well as extreme drought, desertification, and fluctuations of humid/dry cycles) represents the most important agroecosystem factor influencing the life cycle stages of fungi and their ability to colonize crops, survive, and produce toxins. The ability of mycotoxigenic fungi to respond to Climate Change (CC) may induce a shift in their geographical distribution and in the pattern of mycotoxin occurrence. The present review examines the available evidence on the impact of CC factors on growth and mycotoxin production by the key mycotoxigenic fungi belonging to the genera *Aspergillus*, *Penicillium*, and *Fusarium*, which include several species producing mycotoxins of the greatest concern worldwide: aflatoxins (AFs), ochratoxins, and fumonisins (FUMs).

## 1. Introduction

According to the Sixth Assessment Report of the Intergovernmental Panel on Climate Change, Earth’s climate is undergoing adverse global changes as an unequivocal result of anthropogenic activity. The planet’s climate has been going through a period of significant changes since the mid-twentieth century, as demonstrated by the rise in global air and ocean temperatures, the increase in sea level, and the shrinking ice sheets [1]. It is very likely that human activities, especially the large-scale clearing of forests and emission of greenhouse gasses (GHGs) and particulate matter, have been the driver of the observed climate change (CC). Carbon dioxide emissions into the atmosphere due mainly to the burning of fossil fuels are increasing more than 250 times faster than they did from natural sources, leading to an increase in the Earth’s average surface temperature of about 1.18 °C since the late 19th century. As a consequence of this current warming trend, Antarctica lost about 148 billion tons of ice per year between 1993 and 2019, the global sea level rose about 20 cm, and the ocean showed a warming of more than 0.33 °C [2]. Based on scientific evidence, global climate change is projected to continue with a temperature rise of about 1.4 to 5.5 °C over the next century and atmospheric concentrations of CO_2_ are expected to double or triple (from 350–400 to 800–1200 ppb) in the next 25–50 years [1].

In a future in which GHG emissions continue to grow, scientists forecast longer lasting frost-free and growing seasons, increased heavy precipitation events, more intense heat waves, and reduced soil moisture, which in turn might have a profound impact on agriculture. Cultivations are highly sensitive to CC, which could directly affect crop and livestock productivity as well as food security [3]. The occurring environmental changes are slowly shaping the balance between plant growth and related fungal diseases. Climate (temperature, available water, and light quality/quantity; as well as extreme drought, desertification, and fluctuations of humid/dry cycles) represents the most important agroecosystem factor influencing the life cycle stages of fungi and their ability to colonize crops, survive, and produce toxins [4]. Considering the significant implications mycotoxins have for human and animal health, there has been a recent focus on the effect of interactions between environmental factors; however, obtaining a clear picture of the exact effect of the current and future global change in fungal ecology is difficult. The ability of mycotoxigenic fungi to respond to CC may induce a shift in their geographical distribution and in the pattern of mycotoxin occurrence. Indeed, global warming will not only increase the number of crops damaged by insects and, therefore, render them more susceptible to mold infection, but it also might alter the diversity of diseases invading crops; certain fungi could disappear from an environment and appear in new regions previously considered safe, along with the consequent economic and social implications.

Many of the current predictions and hypotheses on the influence of CC on fungal growth and mycotoxin production are based on predictive models using historical or current climatic condition datasets that predominantly consider two-way interactions (temperature X water activity). Very little information is available on the effect of three-way interactions (temperature, water activity (*a_w_*), and CO_2_). The results of the analysis of these models must be interpreted carefully; they should not be considered precise predictions of the future, since they do not consider all of the CC-related abiotic factors and are limited only to certain regions. Furthermore, models developed by various authors are based on in vitro trials and interactions between fungi and input variables [5,6,7,8,9,10,11,12], but they need to be calibrated since in vitro experiments may differ considerably from field conditions where the host plant and the fungi are equally exposed to complex ecological factors [13]. However, Shaw and colleagues demonstrated that the limitations of these models can be overcome by using long-term data sets [14]. In addition, molecular methods have been used to examine the impact of CC, such as the microarray and qPCR approaches, which allow for a better understanding of the relationship between environmental stressors and the expression of specific mycotoxin biosynthetic genes, elucidating the biological and biochemical processes regulating mycotoxin production and the ability of fungi to adapt to environmental stresses. The PCR analysis of the genes encoding mycotoxins by using plant herbarium samples may be another useful approach for identifying trends and factors that affect mycotoxin prevalence over time [15]. There is a need for integrated and interdisciplinary approaches involving agronomists, mycologists, and climate scientists in order to transfer the approaches described above to a global level and support strategies to reduce risk areas and improve public and animal health under predicted future scenarios.

The present review examines the available evidence regarding the impact of CC factors on growth and mycotoxin production (Table 1) by the key mycotoxigenic fungi belonging to the genera *Aspergillus*, *Penicillium*, and *Fusarium*, which include several species producing mycotoxins of greatest concern worldwide: aflatoxins (AFs), ochratoxins, and fumonisins (FUMs).

## 2. Effect of Climate Change on Fungal Distribution

Increased temperatures will observe an overall increase in mycotoxigenic fungi suited to higher temperatures, such as aflatoxin-producing *Aspergillus* species, which represent an important hazard to human and animal health. In fact, since their discovery, while *Penicillium* spp. (OTA and PAT producers) grow and produce mycotoxins mainly in temperate climatic regions, *Aspergillus* spp. (AFs and OTA producers) have been shown to occupy primarily tropical/subtropical regions growing at high temperatures and lowered *a_w_*.

Developing crops are frequently very resistant to infection by *A. flavus* and subsequent AFs contamination unless environmental conditions favor fungal growth and crop susceptibility. Battilani et al. [21] predicted that, within the next century, in a scenario based on +2 °C and +5 °C temperature increase, *A. flavus* will become a food safety issue in maize in central/southern Spain, South Italy, Greece, north/southeast Portugal, Bulgaria, Albania, Cyprus, and Turkey. Paterson et al. [22] predicted that, over the course of the next 100 years, *A. flavus* may outcompete *A. carbonarius*, becoming a greater risk than *ochratoxin* A (OTA). Furthermore, García-Cela et al. [23] indicated that in hotter climatic scenarios *A. niger* may also gain more prevalence over *A. carbonarius*, as the former is better adapted to high temperatures and drier conditions [24] than the latter.

Changes in mycotoxigenic fungi due to CC are already observed. Examples of modified weather regimes impacting mycotoxins were demonstrated by the 2003, 2004, and 2012 summer seasons in Italy, where dry and hot weather (>35 °C) contributed to an outbreak of *A. flavus* on crops, previously uncommon, by outcompeting the more common *Fusarium* species and fumonisins contamination and causing an increase in AFB1 [25,26]. Similarly, in France in 2015, an exceptionally hot and dry year, *A. flavus* was isolated from maize samples, with a percentage equal to 69% [27]. Climatic changes are also expected to modulate the prevalence of mycotoxigenic fungal species in coffee cultivation, with a decline in *Penicillium* species and an increase in AFs-producing *Aspergillus* species [28]. Overall, concerning coffee cultivation, predictions suggest that CC will negatively affect coffee production in terms of both an increase in mycotoxin contamination and the loss of suitable growing areas, which could decrease by about 50% by 2050 [29].

In a study performed by Bellí et al. [30] on the fungi associated with 40 vineyards sampled from four wine-making regions of Spain at three different growth stages in 2002–2003, the highest levels of contamination of black aspergilli were found in grapes from 2003 and from Costers del Segre, the warmest year and the warmest region, respectively. Interestingly, while a positive correlation has been established between the presence of black aspergilli and temperature, no significant correlation has been established with other meteorological factors, such as relative humidity and rainfall. Likewise, Monda et al. [31] recorded a higher occurrence of *A. flavus* in soils from the eastern region of Kenya, characterized by hotter and drier conditions compared to the western region. These findings are consistent with other studies that reported a higher prevalence of *Aspergillus* spp. in drier areas of Makueni compared to humid regions [32]. On the contrary, Baazeem [33] did not obtain significant differences in relative growth rates of *A. flavus* between existing (30 °C, 400 ppm CO_2_, and no drought stress) and future (34 °C, 1000 ppm CO_2_, and drought stress) climate-related factors either in vitro on the pistachio nut agar (PNA) medium or in situ when colonizing raw pistachio nuts.

## 3. Effect of Climate Change on Mycotoxin Contamination

### 3.1. Aflatoxins

Aflatoxins are one of the most toxic mycotoxins known. The dominant aflatoxin produced (AFB1) is the most powerful naturally occurring carcinogen, classified as group I by the International Agency for Research in Cancer [34]. Hence, it is of particular importance to understand how levels of this mycotoxin may shift with the CC that agriculture will experience.

Aflatoxins are produced in different crops by several species of *Aspergillus*, predominantly *A. flavus* and *A. parasiticus*, both characterized by the ability to persist in the most extreme climate warming conditions, as highlighted by their high optimum temperature [22]. Klich [35] reported that the optimum temperature for AFs production by *A. flavus* varies between 24 and 30 °C. A higher optimum temperature (32 °C) was reported by Sorenson et al. [18] on rice grains. A positive correlation between Afs contamination and rain has been shown by Jaime-Garcia and Cotty [36]. In particular, the authors revealed that higher precipitation in the Coastal Bend and the Upper Coast than in the Rio Grande Valley in South Texas from 1997 to 2001 resulted in a more frequent AFs contamination in the first ones, while the Rio Grande Valley showed consistently low AFs levels. A continuous increase in AFs production with increasing *a_w_* in the range of 0.82–0.92 was observed by Mousa et al. [10] in inoculated brown and polished rice. To examine the impact of *a_w_* on AFs production, Zhang et al. [37] used a transcriptomic approach; the authors observed an extensive transcriptomic response during *a_w_* variation between freely available water and water stress, reporting increased AFB1 biosynthesis at 0.99 rather than 0.93 *a_w_*.

With the aim of deeply understanding how these environmental fluctuations affect mycotoxin production, a transcriptomic approach has been used by several researchers who revealed that increased global temperatures, drought stress, and CO_2_ levels have a measurable impact on molecular events in *A. flavus* [38,39,40]. Studies in vitro and on stored maize grain showed that AFB1 production was significantly stimulated under the three-way interacting CC-related factors compared to existing conditions (30 vs. 37 °C; 350–400 vs. 1000 ppm CO_2_; 0.99 vs. 0.93 *a_w_*), as demonstrated by the increase in the relative expression levels of structural and regulatory biosynthetic genes involved in AFs production [39,40]. Further transcriptomic analysis corroborated these findings, showing important regulatory shifts for some of the identified secondary metabolite gene clusters (AFs and cyclopiazonic acid), regulators, sugar transporters, and other stress-related gene clusters under CC-related conditions [38,39]. In contrast, O’Brian et al. [41] reported a reduction in AF production-related genes at 37 °C. Likewise, in a study performed by Schmidt-Heydt et al. [19], two regulatory genes *aflR* and *aflS* were expressed at lower levels at temperatures above 37 °C, resulting in the inhibition of AF synthesis. This is supported by the work of Yu et al. [42], who used RNA-Seq technology to evaluate the expression of genes involved in AF production at 30 °C and 37 °C. They reported an almost total suppression of AF production at 37 °C, with a 50% increase in the expression of AF biosynthesis genes at 30 °C compared to 37 °C. Moreover, in another study, the effect of temperature (35 °C vs. 37 °C), water stress (0.93–0.98 *a_w_*), and CO_2_ (400 vs. 1000 ppm) on AFB1 production was evaluated and stimulation was obtained at 35 °C, 1000 ppm, and 0.98 *a_w_*, while at 37 °C, AFB1 production mostly decreased. However, differential effects were observed, depending on the interacting conditions of the three abiotic factors [33].

While AF contamination until 2004 was mainly confined to imported foods, with only a small percentage of food and feed samples presenting AFB1 concentrations above the regulatory limits [43]; more recently, there has been a widespread incidence of AF contamination in countries not previously considered at risk resulting from persistent drought conditions and rising temperatures [44,45]. In particular, a survey conducted by the European Food Safety Authority (EFSA) established the emerging issue of potential AF contamination in areas of Southern Europe in maize, wheat, and rice linked to the subtropical climate and the numerous hot and dry seasons that have occurred in the last years [46]. In addition, a shift in traditional occurrence areas of AFs is expected. Based on the predictive AFLA-maize and AFLA-wheat models developed by Battilani et al. [21], in a future +2 °C CC scenario, the risk of AF contamination in maize could significantly increase, mainly in areas such as Eastern Europe, the Balkan Peninsula, and the Mediterranean regions, whereas the predicted impact of contamination in wheat was negligible. As a result of maize’s increased AFB1 contamination, Van der Fels-Klerx et al. [47] suggested an increase (up to 50%) of AFM1 occurrence in milk by 2030. Indeed, when AFB1 occurs in feed and is consumed by dairy cattle, it is converted into AFM1, which is excreted in dairy products, such as milk, posing a serious risk to human health [48,49].

The first confirmation of the predicted risk increase in AF occurrence came from Italy and Serbia, where high levels of AFs were found in maize for feed in 2003–2016 as a result of extreme climatic conditions, such as a serious drought and hot summer temperatures [50,51,52]. In a study aimed at assessing post-harvest mycotoxin contamination in Romania in 2012–2015, Gagiu et al. [53] found major contamination in the dry years of 2012 and 2013, with a higher incidence of AFs in the dry areas of Moldavia, the Southern Plain, and Dobrogea, while the incidence was sporadic in the cold and humid areas of Transylvania and in the Southern Hilly Area, where the agroclimatic conditions are less favorable to AF production. However, pluvial precipitation may play a key role in mycotoxin contamination. Higher rainfall resulted in high levels of AFB1 in Southeast, East, and Central Asia in 2017, as well as in India in 2006–2007 [54,55].

### 3.2. Ochratoxin A

Ochratoxin A (OTA) is produced by *Penicillium verrucosum* and several *Aspergillus* species, including *A. ochraceus*, *A. alliaceus*, *A. carbonarius,* and *A. niger*. This mycotoxin has been found on a variety of crops, such as barley, grapes, rye, wheat, and coffee [56]. An essential condition for OTA production is the availability of water. An *a_w_* > 0.95 is considered too humid and can favor other fungi, including yeast, which may limit OTA-producing fungi colonization; an *a_w_* < 0.80 is considered too dry and OTA-producing fungi are unable to produce the mycotoxin. In a study on maize grains, at 30 °C, the production of OTA by *A. ochraceus* was significantly higher at 0.95 than at 0.99 *a_w_* [57]. Alternatively, on barley grains, the optimal growth and toxin production were registered at 30 °C and 0.99 *a_w_* [58]. High humidity has been shown to favor OTA production also at lower temperatures. In vines and grapes, OTA production increased at temperate temperatures (20 °C) and 0.96–0.98 *a_w_* [16,59,60]. In green coffee, Pardo et al. [61] reported that growth and OTA production from *A. ochraceus* were influenced by temperature and *a_w_*; a temperature of 30 °C and *a_w_* of 0.95–0.99 constitute ideal growth conditions for this species, while the maximum production of OTA was observed at 20 °C and 0.99 *a_w_*. Similarly, maximum OTA production by *A. carbonarius* isolates from wine grapes in Greece was observed at 15–20 °C and 0.93–0.96 *a_w_* [62].

Currently, OTA is the most commonly reported mycotoxin in coffee, occurring at variable levels while AFs and other mycotoxins occur less frequently [63,64]. In tropical conditions, where coffee cultivation is widespread, *A. ochraceus* is the major source of OTA, with optimum temperatures for production between 25 and 30 °C. *P. verrucosum* grows optimally at lower temperatures and produces OTA at 25 °C [65]. Considering that temperatures in coffee-producing municipalities have risen by about 0.25 °C per decade since 1974, it is likely that *P. verrucosum* will become less prominent in the future climate scenario [66]. Based on the Index of Dominance developed by Magan et al. [20], the predicted climate shifts will also be unsuited for OTA production by *A. ochraceus* while *A. flavus* will be well suited to cope with future conditions, which could mean that AFs may become the most dominant mycotoxin.

Furthermore, OTA is an important threat in wine. Its presence in grapes is strongly influenced by climatic conditions and, in this regard, several studies have been performed to elucidate the effect of different conditions such as water availability, temperature, and CO_2_ levels on mycotoxin production. Cervini et al. [67,68] showed that *A. carbonarius* growth rate and OTA production were higher at temperature condition simulations (18/31 °C) compared to the climate change scenarios (20/37 °C). In correlation to the higher mycotoxin production, an overall upregulation of the genes involved in OTA biosynthesis was also observed at 18/31 °C. Moreover, in support of this, Oueslati et al. [69] showed that the growth of *A. carbonarius* strains isolated from Tunisian grapes was significantly enhanced at 20/30 °C compared with growth at 20/37 °C and it was even slower at 25/42 °C. A reduction in *A. ochraceus* and *A. carbonarius* growth rates and OTA production following an increase in temperature was also evidenced by Garcia-Cela et al. [70]. Based on these studies, the possible predicted increase in the temperatures may result in a reduction in *A. carbonarius* and OTA production in grapes. However, temperatures will become more suitable for the thermotolerant aspergilli and OTA might be superseded by more dangerous mycotoxins, such as AFs [26]. Contrary results were obtained by Akbar et al. [71], who demonstrated that OTA production was stimulated by CC-related interacting factors for *A. westerdijkiae*, both in vitro and in situ.

### 3.3. Fumonisins

*Fusarium* is a genus that includes plant-pathogenic fungi responsible for a variety of diseases on several different crops [72] and with known potentials in producing mycotoxins capable of well-described adverse effects in humans and animals [73]. *Fusarium* species can tolerate a wide range of temperatures and pH levels [74,75], require a relatively high *a_w_* for growing, are usually well established in a crop before harvesting, and may cause problems in grains following a late harvest after a rainy summer [76].

The change of climate until 2050 in the north of Europe, expected to be milder and more humid, could influence *Fusarium* distribution [77,78], favoring, for example, an increase in *F. graminearum* in central and north Europe [77]. 

Some studies have tried to mimic future growth conditions for mycotoxigenic fungi such as *Fusarium* spp. in the case of weakly raised CO_2_ concentrations together with temperature and water availability [14,79]. Elevated CO_2_ level seems to increase susceptibility to *F. verticillioides* in maize, resulting in 2.5 times *Fusarium* biomass production in specific conditions [80,81] with no impact on mycotoxin levels [80]. 

Fumonisins (FUMs) are mycotoxins produced in cereals by *Fusarium verticillioides* and *Fusarium proliferatum* and related species [82]. FUMs contamination is strictly associated with agroclimatic conditions [83] and is most evident in maize and maize sub-products if compared with other grains and related derivatives [83]. 

FUMs are 15 mycotoxins classified in four different groups, A, B, C, and P, and fumonisin B1 (FB1) is considered the most toxic and abundant, together with fumonisin B2 (FB2) and B3 (FB3) [83].

In temperate and Mediterranean regions, FUMs are prevalent [83,84] and multiple factors are involved and have an impact on FUMs production, such as agronomic, climatic, and environmental factors, where *a_w_* and temperatures play an important role either on the toxins or on *Fusarium* [17,84,85,86]. The growth of *F. verticillioides* occurs at a minimum temperature of 4 °C and has a most favorable temperature of 25 °C [87], while FUMs production occurs between 15 and 25 °C [88]. FUMs growth is strictly related to weather conditions [86] where flowering and the pre-harvesting period are considered crucial [86], and recent climate variability influences their distribution, as reported in Europe, with an increase observed in Central Europe [89,90].

## 4. Conclusions

In the past century, the rates of CC have been registered; although comprehensive and continuous updates have been provided on its potential effects, it is clear that there is currently a significant knowledge gap and only generalizations can be made. Overall, the evidence suggests that CC will negatively affect crops worldwide in terms of loss of suitable cultivation areas and an increase in mycotoxin contamination. Global warming will make growing crops in some areas impossible and, where growing crops will be possible, plants will be subjected to suboptimal climatic conditions, resulting in increased susceptibility to fungal contamination. Furthermore, warmer climates will favor thermotolerant species, leading to the prevalence of *Aspergillus* over *Penicillium* species. Further studies should also focus on the impact of the interacting environmental factors at an epigenetic level, with the aim to integrate these findings with transcriptomic analysis, ecology, and mycotoxin production. Finally, there is a need for conducting studies on other regulated and non-regulated mycotoxins, as well as in other crops and countries to obtain a more comprehensive view of the effects related to CC.

## Figures and Tables

**Table 1:** Effect of temperature and/or water activity on mycotoxin production by *Aspergillus, Penicillium*, and *Fusarium* species.

Fungal Species	Mycotoxin	Temperature °C	*a_w_*	Time (Days)	µg/g	Reference
*A. carbonarius*	OTA	28	0.94	15	0.0314	[16]
			0.96	15	0.126	“
			0.98	15	0.862	“
		25	0.94	15	0.122	“
			0.96	15	0.326	“
			0.98	15	2	“
		20	0.94	15	0.324	“
			0.96	15	1.028	“
			0.98	15	2.743	“
*A. flavus*	AFB1	40	-	21	0	[17]
			0.90	9	0	[9]
		37	-	3	0.7	[18]
			-	5	0.4	“
			-	7	0.3	“
			0.90	9	3.96	[9]
			0.95	9	2.68	“
			0.99	9	2.42	“
		35	-	21	0.001	[17]
			0.90	5	0.0046	[19]
		34	-	3	29	[18]
			-	5	36	“
			-	7	18	“
		30	-	21	0.02	[17]
			0.95	5	3.016	[19]
			0.99	5	2.758	“
		32	-	3	633	[18]
			-	5	760	“
			-	7	760	“
		28	-	2	184	“
			-	4	760	“
			-	7	760	“
		25	-	21	0.060	[17]
			0.90	5	0.0036	[19]
			0.95	5	0.830	“
			0.99	5	1.957	“
			-	4	304	[18]
			-	5	507	“
			-	7	449	“
		20	-	21	0.062	[17]
		18	-	6	34	[18]
			-	9	124	“
		15	-	8	0.3	“
			-	14	0.9	“
			-	21	2	“
			-	21	0.028	[17]
		10	-	21	0.006	“
		11	-	14	<0.01	[18]
			-	21	0.1	“
		8	-	21	<0.01	“
	AFB2	37	-	3	ND	“
			-	5	ND	“
			-	7	ND	“
		34	-	3	0.8	“
			-	5	3	“
			-	7	2	“
		32	-	3	166	“
			-	5	133	“
			-	7	125	“
		28	-	2	20	“
			-	4	167	“
			-	7	111	“
		25	-	4	53	“
			-	5	80	“
			-	7	85	“
		18	-	6	4	“
			-	9	19	“
		15	-	8	0.02	“
			-	14	0.1	“
			-	21	0.2	“
		11	-	14	<0.01	“
			-	21	ND	“
		8	-	21	ND	“
	AFG1	37	-	3	0.01	“
			-	5	ND	“
			-	7	ND	“
		34	-	3	0.6	“
			-	5	0.2	“
			-	7	<0.2	“
		32	-	3	71	“
			-	5	64	“
			-	7	46	“
		28	-	2	64	“
			-	4	458	“
			-	7	180	“
		25	-	4	198	“
			-	5	256	“
			-	7	235	“
		18	-	6	37	“
			-	9	160	“
		15	-	8	0.06	“
			-	14	1	“
			-	21	3	“
		11	-	14	<0.01	“
			-	21	0.09	“
		8	-	21	<0.01	“
	AFG2	37	-	3	ND	“
			-	5	ND	“
			-	7	ND	“
		34	-	3	<0.01	“
			-	5	<0.01	“
			-	7	<0.01	“
		32	-	3	11	“
			-	5	10	“
			-	7	5	“
		28	-	2	10	“
			-	4	56	“
			-	7	25	“
		25	-	4	25	“
			-	5	31	“
			-	7	32	“
		18	-	6	3	“
			-	9	12	“
		15	-	8	<0.01	“
			-	14	<0.01	“
			-	21	0.3	“
		11	-	14	<0.01	“
			-	21	ND	“
		8	-	21	ND	“
*Fusarium verticilloides*	FBs	40	-	21	0	[17]
		35	-	21	0.157	“
		30	-	21	0.02	“
		25	-	21	0.199	“
		20	-	21	0.258	“
		15	-	21	0.03	“
		10	-	21	0	“
*P. verrucosum*	OTA	25	0.95	56	3.6	[20]
			0.99	56	0.15	“
		15	0.95	56	1.8	“
			0.99	56	3	“
*P. verrucosum +* *F. culmorum*	OTA	25	0.95	56	0	“
			0.99	56	0	“
		15	0.95	56	0.01	“
			0.99	56	0	“
*P. verrucosum + F. poae*	OTA	25	0.95	56	0	“
			0.99	56	0	“
		15	0.95	56	0.2	“
			0.99	56	0.06	“

ND = Not detected.
